# Silencing of Transposable Elements Mediated by 5-mC and Compensation of the Heterochromatin Content by Presence of B Chromosomes in *Astyanax scabripinnis*

**DOI:** 10.3390/cells10051162

**Published:** 2021-05-11

**Authors:** Patrícia Barbosa, Zelinda Schemczssen-Graeff, André Marques, Maelin da Silva, Giovani Marino Favero, Bernardo Passos Sobreiro, Mara Cristina de Almeida, Orlando Moreira-Filho, Duílio Mazzoni Zerbinato de Andrade Silva, Fábio Porto-Foresti, Fausto Foresti, Roberto Ferreira Artoni

**Affiliations:** 1 Post Graduate Program in Evolutionary Genetics and Molecular Biology, Department of Genetics and Evolution, Federal University of São Carlos, Rodovia Washington Luís Km 235, São Carlos 13565-905, SP, Brazil; pbarbosa.bio@gmail.com (P.B.); omfilho@ufscar.br (O.M.-F.); 2 Post Graduate Program in Evolutionary Biology, Department of Structural, Molecular and Genetic Biology, State University of Ponta Grossa, Avenida Carlos Cavalcanti 4748, Ponta Grossa 84030-900, PR, Brazil; zelinda1985@hotmail.com (Z.S.-G.); maesilva@uepg.br (M.d.S.); almeidamara@uol.com.br (M.C.d.A.); 3 Department of Botany, Federal University of Pernambuco, Recife 50670-901, PE, Brazil; andre.marques@arapiraca.ufal.br; 4 Department of General Biology, State University of Ponta Grossa, Avenida Carlos Cavalcanti 4748, Ponta Grossa 84030-900, PR, Brazil; gmfavero@yahoo.com.br; 5 Department of Medicine, State University of Ponta Grossa, Avenida Carlos Cavalcanti 4748, Ponta Grossa 84030-900, PR, Brazil; bsobreiro@hotmail.com; 6 Department of Structural and Functional Biology, Institute of Biosciences at Botucatu, Sao Paulo State University (UNESP), Botucatu 18618-689, SP, Brazil; duiliozerbinato@gmail.com (D.M.Z.d.A.S.); f.foresti@unesp.br (F.F.); 7 Faculty of Sciences, Sao Paulo State University (UNESP), Bauru 01049-010, SP, Brazil; fp.foresti@unesp.br

**Keywords:** epigenetics, transposable elements, regulation, methylation, isochromosome

## Abstract

The way in which transcriptional activity overcomes the physical DNA structure and gene regulation mechanisms involves complex processes that are not yet fully understood. Modifications in the cytosine-guanine sequence of DNA by 5-mC are preferentially located in heterochromatic regions and are related to gene silencing. Herein, we investigate evidence of epigenetic regulation related to the B chromosome model and transposable elements in *A. scabripinnis*. Indirect immunofluorescence using anti-5-mC to mark methylated regions was employed along with quantitative ELISA to determine the total genomic DNA methylation level. 5-mC signals were dispersed in the chromosomes of both females and males, with preferential accumulation in the B chromosome. In addition to the heterochromatic methylated regions, our results suggest that methylation is associated with transposable elements (LINE and Tc1-Mariner). Heterochromatin content was measured based on the C-band length in relation to the size of chromosome 1. The B chromosome in *A. scabripinnis* comprises heterochromatin located in the pericentromeric region of both arms of this isochromosome. In this context, individuals with B chromosomes should have an increased heterochromatin content when compared to individuals that do not. Although, both heterochromatin content and genome methylation showed no significant differences between sexes or in relation to the occurrence of B chromosomes. Our evidence suggests that the B chromosome can have a compensation effect on the heterochromatin content and that methylation possibly operates to silence TEs in *A. scabripinnis*. This represents a sui generis compensation and gene activity buffering mechanism.

## 1. Introduction

The way in which transcriptional activity overcomes the physical DNA structure and gene regulation mechanisms involves complex processes that are not yet fully understood. Epigenetic marking, characterized by a heritable and reversible change that regulates gene expression without modifying the DNA sequence [1], such as cytosine methylation, histone modifications, and interference RNA [2], is an open area of research for describing this process. One chemical modification that occurs in the DNA dinucleotide cytosine-guanine sequence (CpG) is 5-methylcytosine (5-mC), where CpG methylation occurs on the fifth carbon of the cytosine [1]. Most CpG dinucleotides (75%) are methylated, corresponding to 1% of total bases. CpG clusters, also called CpG islands, are gene sequences of open chromatin that promote transcription; hence, the cytosines in these sites are not methylated. DNA is methylated by methyltransferase enzymes [3]. While the methylation location is meaningful in heterochromatic regions [4,5], it is also remarkable in fractions of structural genes [6]. In mammals, DNA methylation marks the silencing of gene regions [7]. In this way, the inactivation of transposable elements (TEs) and repetitive elements can be the result of this epigenetic marking. Examples occur in ribosomal DNA sites (rDNA) of the fish *Astyanax janeiroensis* [8,9] or plants of the *Citrus* genus [5]. In this context, transposable elements that initiate their cycle with a small invasion in the genome, followed by an amplification of the number of copies, can lose their motility capacity through mutations (Kidwell and Lish, 2001) or by the action of epigenetic mechanisms [10,11].

DNA methylation is not the only modification responsible for gene regulation in the chromatin condensation level. Eukaryote genomes are packaged in chromatin, represented by the nucleosome and the histone octamer [12]. This packaging occurs during cellular division, altering the chromosomic dynamic [13]. Modifications occurring in the histones affect the chromatin structure directly and consequently affect protein interactions. Such modifications alter the levels of DNA compaction, implying the activation/inactivation of transcriptional genes [14]. They may also aid in the recruitment of chromatin modifiers, such as methyl tags and recognition proteins [12]. The amino acid chains of four histones possess post-translational modification sites (lysine acetylation; mono-, di-, and tri-methylation of lysines; serine phosphorylation, etc.), which have distinct functions of interference in the function and integrity of the genome [15]. DNA methylation and histone methylation act in agreement to maintain the silenced state of the chromatin [13].

B chromosomes are described in many organisms and defined as extra, expendable elements to the genome [16]. The fish *Astyanax scabripinnis* has been presented as a good model for studying B chromosomes [17]. Generally, the B chromosomes of *A. scabripinnis* are partially heterochromatic isochromosomes [18] that suffer auto-paring in meiosis [19] and accumulate families of repetitive DNA, such as *As*51 satellite DNA [20] and the retrotransposon LINE [21]. Nevertheless, the functional aspects of these chromosomes remain an open question. We aimed to investigate evidence of the epigenetic regulation of B chromosomes in the model species *A. scabripinnis*.

## 2. Materials and Methods

### 2.1. Samples and Chromosomic Preparations

We analyzed 62 specimens (28 male and 34 female) of *Astyanax aff. scabripinnis* from the Campos de Jordao region, Sao Paulo State, Brazil, collected with the permission of the Ministério do Meio Ambiente, Instituto Brasileiro do Meio Ambiente e dos Recursos Naturais Renováveis, Instituto Chico Mendes de Conservação da Biodiversidade—ICMBio MMA/IBAMA/SISBIO, number 15115-1: Stream of Fazenda Lavrinha (22°40′49.5″ S and 45°23′31.9″ W). For cytogenetic analysis, specimens were anesthetized with benzocaine 0.01% and dissected. The mitotic chromosomes were obtained from kidney tissue according to Bertollo et al. [22]. C-banding was realized following the protocol described by Sumner [23]. The chromosomes were classified as metacentric (m), submetacentric (sm), subtelomeric (st), and acrocentric (a), as proposed by Levan et al. [24], and organized in the decreasing order of the karyotype. All procedures were undertaken according to the international animal testing protocol and authorized by the Committee of Ethics in Animal Experimentation (protocol number 4509/08) of the State University of Ponta Grossa. The specimens were identified and received a deposit number for the Collection NUPELIA (Núcleo de Pesquisas em Limnologia, Ictiologia e Aquicultura) of the State University of Maringá (number NUP 17482).

### 2.2. Quantification of the Methylation and Heterochromatic Regions

Genomic DNA was extracted from B+ and B− individuals using the CTAB method [25] and purified (Macherey-Nagel GmbH and Co. KG, Bethlehem, PA, USA) according to the manufacturer’s instructions. The total 5-methylcytosine (5-mC) was determined using the 5-mC DNA ELISA kit (Zymo Research Corp., Irvine, CA, USA) according to the manufacturer’s instructions. The kit uses an anti-5-methylcytosine monoclonal antibody that is sensitive and specific to 5-mC. The result was expressed as a percentage of 5-mC in a DNA sample, calculated using a standard curve generated with specially designed controls included in the kit. This method was used to compare levels of 5-mC between male and female DNA, with the presence and absence of supernumerary chromosomes.

The quantification of heterochromatin (HT) was measured in 32 metaphases, 16 B+ (eight males; eight females) and 16 B− (eight males; eight females), using ImageJ software (US National Institutes of Health, Bethesda, MD, USA). The length of heterochromatin blocks (LHB) was measured in relation to the total length of the higher chromosome (first pair metacentric) of the complement (LCC), where rate HT = LHB/LCC.

### 2.3. Statistical Analysis

The mean comparison between groups was realized by analysis of variance (ANOVA) using Tukey’s post hoc test after normalization of data by logarithmic transformation. The alpha error established for statistical significance was 5%.

### 2.4. Immunodetection of Methylated DNA, Chromosome Probe, and Fluorescence In Situ Hybridization (FISH)

Immunoassay of 5-methylcytosine (Eurogentec, cat No. MMS-900P-A) was performed according to Ruffini-Castiglione et al. [26]. For sequential hybridization of the *As*51 satellite DNA sequence, the following protocol for washing and removing the antibody was established: washing and stabilization with PBS 1× for 10 min, washing in 4 × SSC/0.05% Tween at room temperature for 10 min, and alcoholic series 70%, 80%, and 90% for 5 min each.

The satellite DNA *As*51 described by Mestriner et al. [20] was obtained from the nuclear DNA of *A. scabripinnis* using the primers Fw 5′-GGTCAAAAAGTCGAAAAA-3′ and Rv 5′-GTACCAATGGTAGACCAA-3′ during 35 cycles of amplification in an Eppendorf Mastercycler (Eppendorf Corporate, Hamburg, Germany)(1 min at 95 °C, 45 s at 56 °C, 1 min at 72 °C, and 5 min at 72 °C). The element Tc1-Mariner [27] was obtained using the unique primer 5′-CACTCACCGGCCACTTTATTA-3′ during 35 cycles of amplification (1 min at 94 °C, 50 s at 62.5 °C, 2 min at 72 °C, and 5 min at 72 °C). The PCR was conducted in a 25-microliter reaction mix with 0.4 µM of primer, 200 ng of DNA template, 0.2 mM of dNTPs, 1× reaction buffer, 2.0 mM of MgCl_2_, and 2U of Taq Polymerase (Invitrogen, Waltham, MA, USA). The element LINE [21] was obtained from the nuclear DNA of *A. scabripinnis* using the primers Fw 5′-CAGTGTGCATCTGATTGTGT-3′ and Rv 5′-CGCAGACGCTTTTATCCA-3′ during 35 cycles of amplification in the Eppendorf Mastercycler (1 min at 95 °C, 45 s at 56 °C, 1 min at 72 °C, and 5 min at 72 °C).

The satellite DNA probe *As*51 and the elements Tc1-Mariner and LINE were labeled with Nick translation using biotin 14-dATP (Bionick Labeling System, Invitrogen, Waltham, MA USA) and digoxigenin 16-dUTP (Dignick mix, Roche, Mannheim, Germany) in a dry bath (Loccus Biotecnologia, São Paulo, SP, Brazil) (1 h 30 min at 65 °C and 15 min at 15 °C), respectively.

FISH was performed using the protocol of Pinkel et al. [28], adapted for fish in high stringency conditions (2.5 ng/mL probe, 50% deionized formamide, 10% sulfate dextran, 2 × SSC at 37 °C overnight). After hybridization, slides were washed in 15% formamide/0.2 × SSC at 42 °C for 20 min and 4 × SSC/0.05% Tween at room temperature for 10 min. The *As*51 probe was detected using Alexa Fluor 488 streptavidin (Molecular Probes^TM^). Anti-digoxigenin-rhodamine (Roche, Mannheim, Germany) was used to detect the Tc1-Mariner element probe. The chromosomes were counterstained with DAPI (0.2 µg/mL) diluted in antifading solution (Fluka, Mannheim, Germany). The metaphases were analyzed using epifluorescence microscopy (Zeiss AxioCam MRm and ZEN Pro 2011 software (version 1.1.0.0, Carl Zeiss, Oberkochen, Germany).

### 2.5. Sequencing and Characterization of the Tc1-Mariner Element Obtained Fragment

The amplification products for the Tc1-Mariner element were separated in 1% agarose gel, and the DNA bands were purified using a PCR DNA and Gel Band Purification Kit (GE Healthcare, Chicago, IL, USA) according to the manufacturer’s instructions. The sequences were linked to the plasmid pGEM^®^-T Easy Vector Systems (Promega, Madison, WI, USA) and transformed into cells of competent *Escherichia coli* DH5α using CaCl_2_ treatment. Recombinant plasmids were extracted by mini-preparation using the Illustra plasmidPrep Mini Spin Kit (GE Healthcare). Nucleotide sequencing of the clones and DNA fragments was performed in an automatic sequencer ABI 3130 × 1 using the Big Dye kit (Applied Biosystems, Foster City, CA, USA) according to the manufacturer’s instructions. The sequences were aligned in the Clustal W program [29], using the BioEdit v7.0 editor [30]. To verify their identity, the sequences were subjected to a search against the database of Dfam (https://dfam.org/search/sequence accessed on 5 May 2021) [31] and then deposited on GenBank (http://www.ncbi.nlm.nih.gov/genbank/ accessed on 5 May 2021) with the following accession number: MT038010.

## 3. Results

A modal diploid number of 2*n* = 50 chromosomes was confirmed for *A. scabripinnis* with a polymorphic occurrence of an additional B chromosome.

**Figure 1:**
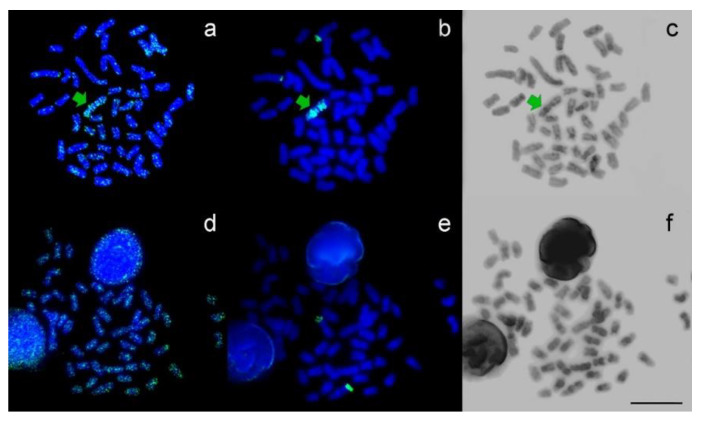
Metaphases of B+ (**a**–**c**) and B− (**d**–**f**) *Astyanax scabripinnis* sequentially treated with anti-5-mC (**a**,**d**), FISH with an *As*51 probe (**b**,**e**), and localization of heterochromatin regions using the C-banding technique (**c**,**f**). The B chromosome is indicated by green arrow It is possible to observe an evident marking in the telomeric region of another chromosomal pair, both in the B+ and B− genomes, strongly marked by *As*51 probes (**b**,**e**). One of the pairs of complement A is probably related to the origin of the B chromosome of *A*. *scabripinnis*. Bar = 10 µm.

The 5-mC signals showed that it dispersed in mitotic metaphase chromosomes in both females and males. Additionally, the B chromosome evidenced preferential accumulation of 5-mC (Figure 1a,d). After 5-mC detection, the slides were sequentially submitted to fluorescent in situ hybridization with the *As*51 satellite DNA probe (Figure 1b,e) and C-banding (Figure 1c,f).

The results indicate that both the location of the satellite DNA and the heterochromatic regions are not exclusively associated with methylation regions. The *As*51 satellite DNA was located in an acrocentric chromosomic pair and a pericentromeric region in both arms of the B chromosome, while the heterochromatic regions were located in pericentromeric regions of most chromosomes and in the distal region of pair one, while the B chromosome was partially heterochromatic.

**Figure 2:**
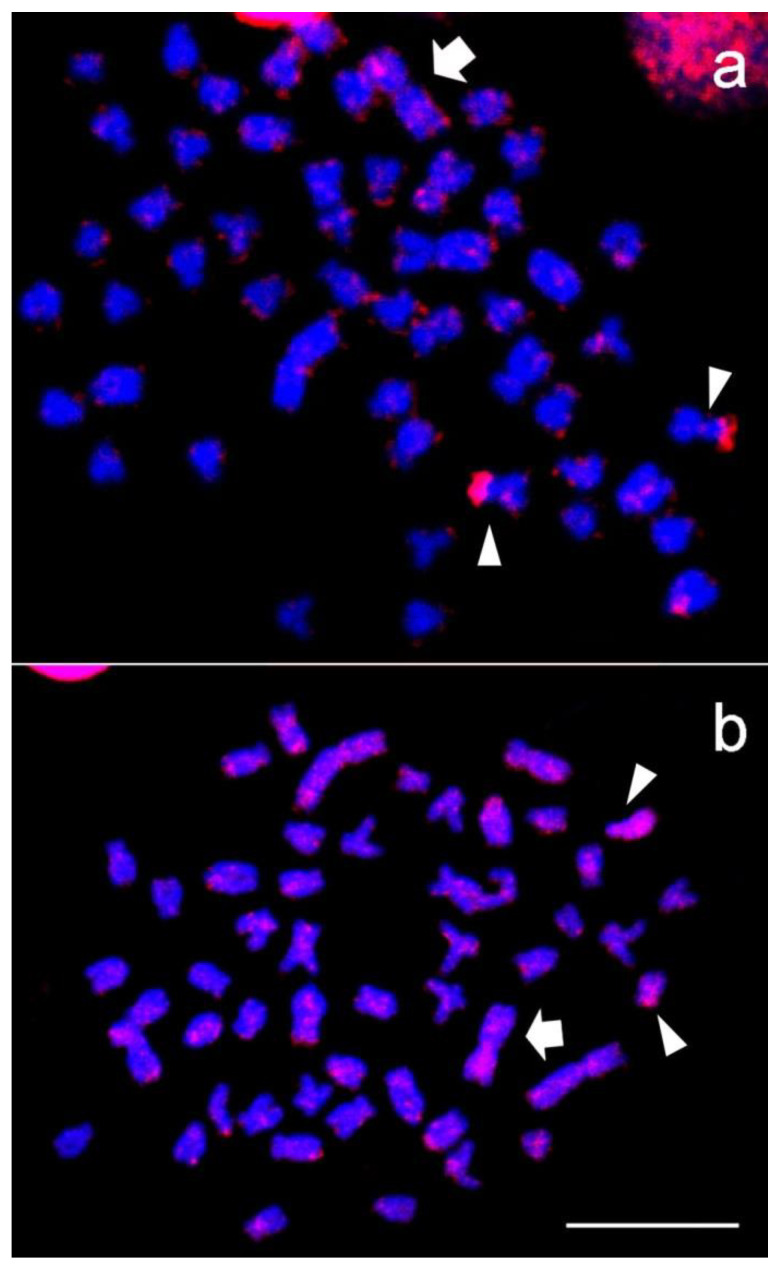
Chromosome mapping with the probes Tc1-Mariner (**a**) and LINE (**b**) in *Astyanax scabripinnis*. B chromosomes are indicated by the big arrows. Tc1-Mariner presents preferential accumulation in the short arm of a medium metacentric pair (little arrow) and small blocks in the other complement chromosomes. LINE has a pattern of localization dispersed in the other chromosomes, with strong accumulation along a pair of complement submetacentric chromosomes (arrows). Bar = 10 µm.

FISH revealed a dispersed pattern of localization of the Tc1-Mariner transposable element between the A and B chromosomes, as well as an accumulation in the short arm of metacentric pair 2 and blocks concentrated in the short arm of a medium metacentric pair (Figure 2a). The in situ localization of LINE was scattered in all chromosomes of the standard complement and in the B chromosome, with preferential accumulation along a pair of complement submetacentric chromosomes (Figure 2b).

**Figure 3:**
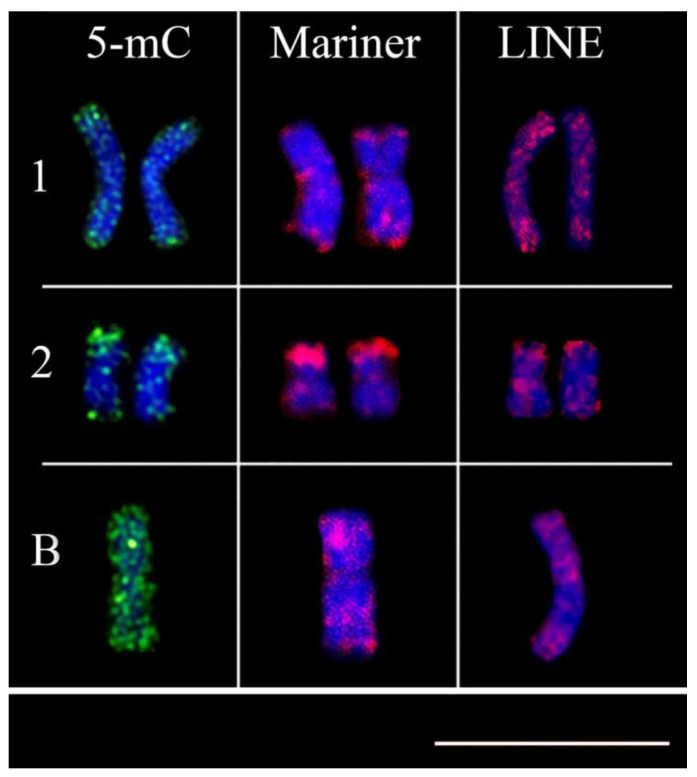
Association of immunodetection of methylated DNA and transposable elements. n the first line, chromosome pair 1, highlighted in the second line, the metacentric chromosome pair, and finally B chromosome, all marked by 5mC, Tc1-mariner and LINE, respectively Bar = 10 µm.

In contrast with the 5-mC and in situ hybridization, the indirect immunofluorescence of the Tc1-Mariner and LINE probes suggests that the transposable element regions are methylated (Figure 3).

**Figure 4:**
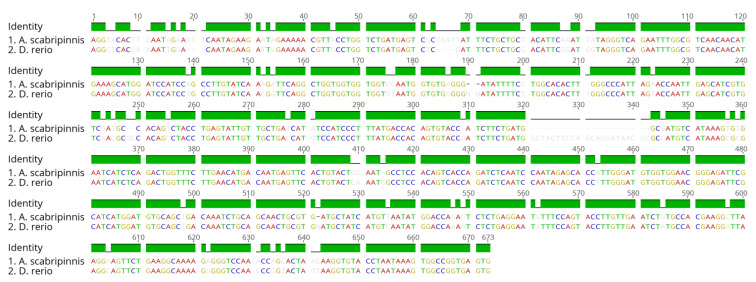
Alignment of the Tc1-Mariner transposon sequences of *A*. *scabripinnis* and *D*. *rerio*, showing a pairwise identity of 87%. The alignment was performed using Geneious v10.2.6.

A partial sequence of the Tc1-Mariner transposon was obtained from the *A. scabripinnis* population in the present study. The obtained nucleotide sequence was submitted to the Dfam sequence search (https://dfam.org/family/DF0003847/features accessed on 5 May 2021) and showed about an 87% similarity with the Tc1-Mariner DNA transposon of the *Danio rerio* (Figure 4).

The quantification of 5-mC levels in the genomic DNA and total heterochromatin (TH) was not significantly different between individuals (Table 1 and Table 2). Individual data can be found in tables in the Supplementary Materials (Tables S1 and S2). The mean percentage of 5-mC in male individuals without extra chromosomes was 10.7%, and the mean quantity of TH was 23.9. For individuals carrying the B chromosome, these values were 13.3% (5-mC) and 25.8 (TH).

**Figure 5:**
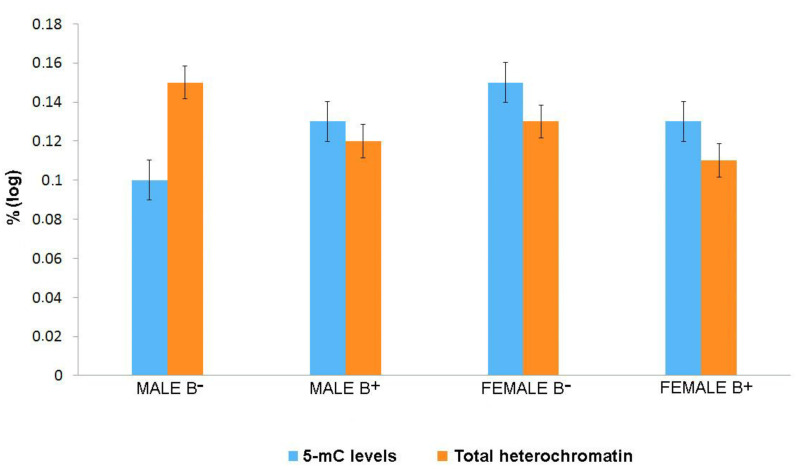
Bar plot showing the methylation conditions of the total genomic DNA (**blue**) and heterochromatin content (**orange**). Genome of male and female without B chromosome is represented by B^−^ and genome with B chromosome by B+. The data were normalized on a logarithmic scale.

Females without B chromosomes had higher average values than males, with 15.3% (5-mC) and 22 (TH). The averages for females with the extra chromosome were 13.9% (5-mC) and 23.3 (TH). Results for 5-mC and for TH are shown in Figure 5.

## 4. Discussion

The total heterochromatin content and the DNA methylation levels did not differ between male and female cells, with and without B chromosomes in *Astyanax scabripinnis*. However, the amount of TE in B− and B+ genomes shows a difference, since the B chromosome behaves as a deposit of moving elements. This likely represents a compensation mechanism and TE silencing.

The B chromosomes of *A. scabripinnis* were composed of a greater amount of heterochromatin in the pericentromeric region of the short and long arms [18,19,20] and this study. Thus, individuals with B chromosomes would be expected to have a higher heterochromatin content when compared to individuals without them. However, our results show that the presence of a B chromosome in the karyotype did not alter the heterochromatin quantity found in the genome (*p* = 0.022). We suggest that there is possible compensation for the total content of heterochromatin between B chromosome-carrying individuals and non-carrier individuals; heterochromatin quantity is broadly distributed in the complement A chromosomes when a B chromosome is absent. Our findings are similar to those of Chumová et al. [32], who analyzed the effect of the presence of a B chromosome on the size of the genome of the grass *Anthoxanthum.* They found that the intraspecific variability of heterochromatin occurring in this genus was caused by complement A chromosomes and not by the presence of B chromosomes.

*Astyanax scabripinnis* is characterized by the formation of small and isolated populations, which facilitates the establishment. This leads to polymorphism and karyotype variability between individuals (revisited by Moreira-Filho et al. [17]). In this scenario, different populations tend to present a marked polymorphism of heterochromatin [33]. Polymorphic chromosome patterns of distribution of heterochromatin also occur in other species of *Astyanax,* including *A. fasciatus* [34], *A. serratus* (cited as *Astyanax* sp. D) [35], and *A. bockmanni* [36]. Nonetheless, the mechanism that generates this diversification in the chromosomic distribution of heterochromatin remains to be investigated.

Constitutive heterochromatin is recognized as a highly stable structure, is transcriptionally inactive, and is comprised of repetitive elements. Yet, recent studies affirm that these regions are highly dynamic, and their maintenance could be related to the transcription and participation of non-coding RNAs, cell aging, or even stress and epigenetic modifications (revisited by Wang et al. [37]). In *Drosophila*, the heterochromatin located next to the genes responsible for eye color causes “white–red” variegation, suggesting heterochromatin’s ability to regulate nearby genes [38].

Utilizing the specific antibody anti-5-mC against metaphases of *A. scabripinnis* showed a dispersed methylation profile between the euchromatic and heterochromatic regions. Indirect immunofluorescence followed by C-banding revealed that methylated DNA is not exclusively associated with heterochromatic regions. This differs from the results obtained by Schmid et al. [39], where hypermethylated chromosomal regions in nine species of fish were confined to constitutive heterochromatin, including the sex chromosomes present in these species, as in mammals [40] and birds [41]. In the context of *A. scabripinnis*, these results indicate that the gene silencing mechanism occurs broadly in the genome, not only in the heterochromatic regions. These data suggest strong epigenetic interference in the genome of *A. scabripinnis.*

The levels of 5-mC obtained by ELISA from the total genome of individuals with and without B chromosomes did not show significant differences in methylation (*p* = 0.02), reinforcing the hypothesis that the B chromosome blocks the gene inactivation in this species when it is present in the genome. DNA methylation patterns were investigated and analyzed through in situ digestion with restriction endonucleases for B chromosomes in the grasshopper *Eyprepocnemis plorans* during various life stages. In this case, although these cells are not methylated, the NOR regions remain inactive, indicating that methylation is not a direct cause of suppression of ribosomal genes in this species [42]. On the other hand, RNA sequences denominated as piRNA that have 25 to 32 nucleotides participate in silencing TEs through post-transcriptional mechanisms and through epigenetic changes. In flies and mice, where the chromatin conformational change mechanism is better understood, the piRISC complex helps to induce methylation patterns in specific loci of DNA from which TEs are expressed [43].

Indirect immunofluorescence and in situ hybridization suggest an association among methylated regions and the localization of the transposable elements LINE and Tc1-Mariner. Transposons and retrotransposons are targets of DNA methylation, a defense of the host genome against proliferation and deleterious consequences of these TEs [44]. However, the methylation of cytosine may not be the only possible cause of silencing [10]. Thus, epigenetic mechanisms are used as a necessary defense to suppress TE activity, making them incapable of producing proteins by silencing chromatin [11]. Although most TEs invade heterochromatic regions [45], physical mapping analyses of LINE [21], and the present study of Tc1-Mariner, demonstrate localization in euchromatic regions, similar to the results obtained by Schemberger et al. [46] in species of Parodontidae. Once TEs scrape off the inactivation and lose their original function, they can suffer a process called “molecular domestication”. This tends to benefit the host genome in establishing new cellular functions, such as control of the cellular cycle, proliferation, apoptosis, and chromatin structure (revisited by Sinzelle et al. [47]).

The co-localization of marking (5-mC, LINE, and Tc1-Mariner) in chromosome pairs 1–2 and the B chromosome of *A. scabripinnis* suggests that these methylated elements are being silenced by the genome. Hypomethylated centromeric regions of the B chromosome in corn become hypermethylated when transferred into oat plants [48]. In the *Citrus* species, the methylation of histone H3 was related to the silencing of retrotransposons, or gene silencing, due to their sparse pattern of accumulation along the euchromatin [5].

We verified a discrete difference in the heterochromatin and DNA methylation content in males and a larger difference in female *Astyanax scabripinnis* without B chromosomes. Both methodologies indicate a possible sex-linked relationship that should be more deeply explored given the absence of reports of morphologically differentiated sex chromosomes in these fish. The lowest level of methylation was found in males without B chromosomes, in relation to females, despite the heterochromatin content of these males being the highest among all possibilities (Figure 5). This raises the hypothesis that heteromorphism is linked to the male sex in these fish.

In conclusion, this evidence suggests that the B chromosome of *A. scabripinnis* can exert a compensation effect on the total content of heterochromatin and that methylation acts to silence the transposable elements in this species. This represents a sui generis compensation and gene activity buffering mechanism.

## Figures and Tables

**Table 1:** Summary of 5-mC levels measured in the genomic DNA of *Astyanax scabripinnis.* Individuals 1 and 2 are male B + and B−, respectively. Individuals 3 and 4 are females, B + and B−, respectively. Confidence interval for mean: *p* > 95%.

	Number of Cells	Mean	Lower Bound	Upper Bound
1	242	0.1273	0.1055	0.1490
2	233	0.1582	0.1316	0.1848
3	241	0.1163	0.0975	0.1352
4	214	0.1390	0.1137	0.1643
Total	930	0.1349	0.1233	0.1465

**Table 2:** Summary of heterochromatin (TH) values measured in chromosomes of *Astyanax scabripinnis*. Individuals 1 and 2 are male B+ and B−, respectively. Individuals 3 and 4 are females, B+ and B−, respectively. Confidence interval for mean: *p* > 95%.

	Number of Cells	Mean	Lower Bound	Upper Bound
1	242	−1.0056	−1.0363	−0.9749
2	233	−0.9478	−0.9857	−0.9100
3	241	−1.0385	−1.0684	−1.0085
4	214	−1.0084	−1.0480	−0.9688
Total	930	−1.0003	−1.0176	−0.9830

## Data Availability

All data are contained within the manuscript, and individual FISH images, tables, and karyotypes are available from the authors on request.

## Supplementary Materials

The following are available online at https://www.mdpi.com/article/10.3390/cells10051162/s1, Table S1: Individual values of 5−mC in the genomic DNA of B+ and B− males, Table S2: Individual values of 5−mC in the genomic DNA of B+ and B− females.
