# Supplementary material for: Silencing of Transposable Elements Mediated by 5-mC and Compensation of the Heterochromatin Content by Presence of B Chromosomes in Astyanax scabripinnis
Source: Cells. 2021 May 11;10(5):1162. doi: 10.3390/cells10051162 (PMC8151356; doi:10.3390/cells10051162)
Supplement: Supplementary file 1 [file cells-10-01162-s001.zip › cells-1190468-supplementary.pdf]

cells

MDPI

Supplementary Materials

Table S1. Individual values of 5−mC in the genomic DNA of B+ and B− males.

	Individual	Value	Log value
Male B−			
	1	0.07	−1.1549019599857431
	1	0.066	−1.1804560644581312
	1	0.057	−1.2441251443275085
	1	0.053	−1.2757241303992111
	1	0.052	−1.2839966563652008
	1	0.052	−1.2839966563652008
	1	0.08	−1.0969100130080565
	1	0.061	−1.2146701649892331
	1	0.105	−0.978810700930062
	1	0.089	−1.0506099933550872
	1	0.107	−0.97061622231479039
	1	0.067	−1.1739251972991736
	1	0.051	−1.2924298239020637
	1	0.046	−1.3372421683184259
	1	0.054	−1.2676062401770316
	1	0.054	−1.2676062401770316
	1	0.059	−1.2291479883578558
	1	0.052	−1.2839966563652008
	1	0.039	−1.4089353929735009
	1	0.047	−1.3279021420642825
	1	0.062	−1.2076083105017461
	1	0.048	−1.3187587626244128
	1	0.108	−0.96657624451305035
	1	0.045	−1.3467874862246563
	1	0.074	−1.1307682802690238
	1	0.079	−1.1023729087095586
	1	0.096	−1.0177287669604316
	1	0.08	−1.0969100130080565
	1	0.047	−1.3279021420642825
	1	0.058	−1.2365720064370627
	1	0.093	−1.031517051446065
	1	1.198	0.078456818053292562
	1	0.177	−0.75202673363819339
	1	0.075	−1.1249387366083
	1	0.047	−1.3279021420642825
	1	0.066	−1.1804560644581312
	1	0.098	−1.0087739243075051
	1	0.151	−0.82102305270683062
	1	0.073	−1.1366771398795441
	1	0.089	−1.0506099933550872
	1	0.14	−0.85387196432176193
	1	0.055	−1.2596373105057561
	1	0.08	−1.0969100130080565

	1	0.103	−0.98716277529482777
	1	0.1	−1
	1	0.072	−1.1426675035687315
	1	0.077	−1.1135092748275182
	1	0.085	−1.0705810742857072
	1	0.083	−1.080921907623926
	1	0.067	−1.1739251972991736
	1	0.089	−1.0506099933550872
	1	0.107	−0.97061622231479039
	1	0.107	−0.97061622231479039
	1	0.146	−0.835647144215563
	1	0.091	−1.0409586076789064
	1	0.152	−0.81815641205522749
	1	0.101	−0.99567862621735737
	1	0.082	−1.0861861476162833
	1	0.141	−0.8507808873446201
	1	0.133	−0.87614835903291421
	1	0.1	−1
	1	0.061	−1.2146701649892331
	1	0.112	−0.9507819773298184
	1	0.075	−1.1249387366083
	1	0.094	−1.0268721464003014
	1	0.064	−1.1938200260161129
	1	0.071	−1.1487416512809248
	1	0.084	−1.0757207139381184
	1	0.056	−1.2518119729937995
	1	0.069	−1.1611509092627446
	1	0.145	−0.83863199776502517
	1	0.066	−1.1804560644581312
	1	0.067	−1.1739251972991736
	1	0.06	−1.2218487496163564
	1	1.258	0.099680641109250123
	1	0.2	−0.69897000433601875
	1	0.097	−1.0132282657337552
	1	0.118	−0.92811799269387463
	1	0.078	−1.1079053973095197
	1	0.077	−1.1135092748275182
	1	0.128	−0.89279003035213167
	1	0.126	−0.89962945488243706
	1	0.069	−1.1611509092627446
	1	0.066	−1.1804560644581312
	1	0.133	−0.87614835903291421
	1	0.219	−0.65955588515988162
	1	0.153	−0.81530856918240124
	1	0.084	−1.0757207139381184
	1	0.084	−1.0757207139381184
	1	0.103	−0.98716277529482777
	1	0.075	−1.1249387366083
	1	0.111	−0.95467702121334252
	1	0.108	−0.96657624451305035
	1	0.076	−1.1191864077192086

	1	0.075	−1.1249387366083
	1	0.093	−1.031517051446065
	1	0.089	−1.0506099933550872
	1	0.086	−1.0655015487564323
	1	0.057	−1.2441251443275085
	1	0.079	−1.1023729087095586
	1	0.059	−1.2291479883578558
	1	0.115	−0.9393021596463883
	1	0.073	−1.1366771398795441
	1	0.072	−1.1426675035687315
	1	0.09	−1.0457574905606752
	1	0.075	−1.1249387366083
	1	0.201	−0.69680394257951106
	1	0.073	−1.1366771398795441
	1	0.129	−0.889410289700751
	1	0.098	−1.0087739243075051
	1	0.078	−1.1079053973095197
	1	0.079	−1.1023729087095586
	1	0.103	−0.98716277529482777
	1	0.152	−0.81815641205522749
	1	0.153	−0.81530856918240124
	1	0.143	−0.84466396253493825
	1	0.135	−0.86966623150499389
	1	0.065	−1.1870866433571443
	1	0.173	−0.76195389687120463
	1	0.079	−1.1023729087095586
	1	0.089	−1.0506099933550872
	1	0.056	−1.2518119729937995
	1	0.065	−1.1870866433571443
	1	0.053	−1.2757241303992111
	1	0.153	−0.81530856918240124
	1	0.162	−0.790484985457369
	1	0.071	−1.1487416512809248
	1	0.078	−1.1079053973095197
	1	0.15	−0.82390874094431876
	1	0.075	−1.1249387366083
	1	0.083	−1.080921907623926
	1	0.038	−1.4202164033831899
	1	0.094	−1.0268721464003014
	1	0.038	−1.4202164033831899
	1	1.222	0.087071205906535415
	1	0.094	−1.0268721464003014
	1	0.09	−1.0457574905606752
	1	0.093	−1.031517051446065
	1	0.107	−0.97061622231479039
	1	0.08	−1.0969100130080565
	1	0.088	−1.0555173278498313
	1	0.135	−0.86966623150499389
	1	0.11	−0.958607314841775
	1	0.109	−0.96257350205937642
	1	0.079	−1.1023729087095586

	1	0.188	−0.72584215073632019
	1	0.078	−1.1079053973095197
	1	0.112	−0.9507819773298184
	1	0.094	−1.0268721464003014
	1	0.077	−1.1135092748275182
	1	0.151	−0.82102305270683062
	1	0.103	−0.98716277529482777
	1	0.126	−0.89962945488243706
	1	0.087	−1.0604807473813815
	1	0.127	−0.89619627904404309
	1	0.071	−1.1487416512809248
	1	0.881	−0.055024091587952087
	1	0.079	−1.1023729087095586
	1	0.203	−0.69250396208678711
	1	0.066	−1.1804560644581312
	1	0.156	−0.80687540164553839
	1	0.13	−0.88605664769316317
	1	0.104	−0.98296666070121963
	1	0.093	−1.031517051446065
	1	0.099	−1.00436480540245
	1	0.106	−0.97469413473522981
	1	0.104	−0.98296666070121963
	1	0.146	−0.835647144215563
	1	0.118	−0.92811799269387463
	1	0.167	−0.77728352885241669
	1	0.078	−1.1079053973095197
	1	0.083	−1.080921907623926
	1	0.104	−0.98296666070121963
	1	0.05	−1.3010299956639813
	1	0.076	−1.1191864077192086
	1	0.125	−0.90308998699194354
	1	0.063	−1.2006594505464183
	1	0.062	−1.2076083105017461
	1	0.12	−0.92081875395237522
	1	0.108	−0.96657624451305035
	1	0.106	−0.97469413473522981
	1	0.708	−0.149966742310231
	1	0.147	−0.83268266525182388
	1	0.137	−0.86327943284359321
	1	0.212	−0.67366413907124856
	1	0.103	−0.98716277529482777
	1	0.174	−0.75945075171740029
	1	0.083	−1.080921907623926
	1	0.091	−1.0409586076789064
	1	0.221	−0.65560772631488928
	1	0.153	−0.81530856918240124
	1	0.124	−0.906578314837765
	1	0.086	−1.0655015487564323
	1	0.128	−0.89279003035213167
	1	0.056	−1.2518119729937995
	1	0.167	−0.77728352885241669

	1	0.079	−1.1023729087095586
	1	0.118	−0.92811799269387463
	1	0.118	−0.92811799269387463
	1	0.137	−0.86327943284359321
	1	0.086	−1.0655015487564323
	1	0.091	−1.0409586076789064
	1	0.066	−1.1804560644581312
	1	0.126	−0.89962945488243706
	1	0.155	−0.8096683018297085
	1	0.156	−0.80687540164553839
	1	0.071	−1.1487416512809248
	1	0.153	−0.81530856918240124
	1	0.125	−0.90308998699194354
	1	0.132	−0.87942606879415008
	1	0.063	−1.2006594505464183
	1	0.035	−1.4559319556497243
	1	0.074	−1.1307682802690238
	1	0.062	−1.2076083105017461
	1	0.119	−0.9244530386074693
	1	1.292	0.1112625136590653
	1	0.075	−1.1249387366083
	1	0.125	−0.90308998699194354
	1	0.087	−1.0604807473813815
	1	0.075	−1.1249387366083
	1	0.049	−1.3098039199714864
	1	0.093	−1.031517051446065
	1	0.083	−1.080921907623926
	1	0.139	−0.85698519974590492
	1	0.15	−0.82390874094431876
	1	0.111	−0.95467702121334252
	1	0.271	−0.56703070912559428
	1	0.091	−1.0409586076789064
	1	0.142	−0.8477116556169435
	1	0.135	−0.86966623150499389
	1	0.062	−1.2076083105017461
	1	0.056	−1.2518119729937995
	1	0.103	−0.98716277529482777
	1	0.076	−1.1191864077192086
	1	0.101	−0.99567862621735737
	1	0.115	−0.9393021596463883
	1	0.099	−1.00436480540245
	1	0.294	−0.53165266958784274
	1	0.09	−1.0457574905606752
	1	0.091	−1.0409586076789064
	1	0.105	−0.978810700930062
	1	0.93	−0.031517051446064863
Male B+			
	2	0.07	−1.1549019599857431
	2	0.098	−1.0087739243075051
	2	0.125	−0.90308998699194354
	2	0.118	−0.92811799269387463

	2	0.159	−0.79860287567954846
	2	0.091	−1.0409586076789064
	2	0.101	−0.99567862621735737
	2	0.097	−1.0132282657337552
	2	0.127	−0.89619627904404309
	2	0.304	−0.51712641639124624
	2	0.146	−0.835647144215563
	2	0.111	−0.95467702121334252
	2	0.075	−1.1249387366083
	2	0.192	−0.71669877129645043
	2	0.105	−0.978810700930062
	2	0.133	−0.87614835903291421
	2	0.099	−1.00436480540245
	2	0.142	−0.8477116556169435
	2	0.071	−1.1487416512809248
	2	0.142	−0.8477116556169435
	2	0.801	−0.096367483915762317
	2	0.072	−1.1426675035687315
	2	0.068	−1.1674910872937636
	2	0.08	−1.0969100130080565
	2	0.103	−0.98716277529482777
	2	0.091	−1.0409586076789064
	2	0.085	−1.0705810742857072
	2	0.103	−0.98716277529482777
	2	0.106	−0.97469413473522981
	2	0.067	−1.1739251972991736
	2	1	0
	2	0.084	−1.0757207139381184
	2	0.188	−0.72584215073632019
	2	0.147	−0.83268266525182388
	2	0.094	−1.0268721464003014
	2	0.069	−1.1611509092627446
	2	0.11	−0.958607314841775
	2	0.195	−0.70996538863748193
	2	0.133	−0.87614835903291421
	2	0.079	−1.1023729087095586
	2	0.1	−1
	2	0.091	−1.0409586076789064
	2	0.129	−0.889410289700751
	2	0.196	−0.707743928643524
	2	0.178	−0.74957999769110606
	2	0.129	−0.889410289700751
	2	0.127	−0.89619627904404309
	2	0.146	−0.835647144215563
	2	0.118	−0.92811799269387463
	2	0.2	−0.69897000433601875
	2	0.105	−0.978810700930062
	2	0.315	−0.50168944621039946
	2	0.266	−0.575118363368933
	2	0.094	−1.0268721464003014
	2	0.085	−1.0705810742857072

	2	0.135	−0.86966623150499389
	2	0.089	−1.0506099933550872
	2	0.124	−0.906578314837765
	2	0.119	−0.9244530386074693
	2	0.075	−1.1249387366083
	2	0.066	−1.1804560644581312
	2	0.12	−0.92081875395237522
	2	0.065	−1.1870866433571443
	2	0.073	−1.1366771398795441
	2	0.098	−1.0087739243075051
	2	0.079	−1.1023729087095586
	2	0.103	−0.98716277529482777
	2	0.857	−0.067019178076801841
	2	0.134	−0.8728952016351923
	2	0.094	−1.0268721464003014
	2	0.049	−1.3098039199714864
	2	0.094	−1.0268721464003014
	2	0.095	−1.0222763947111522
	2	0.103	−0.98716277529482777
	2	0.134	−0.8728952016351923
	2	0.761	−0.11861534322942717
	2	0.201	−0.69680394257951106
	2	0.097	−1.0132282657337552
	2	0.121	−0.91721462968354994
	2	0.169	−0.77211329538632645
	2	0.134	−0.8728952016351923
	2	0.108	−0.96657624451305035
	2	0.131	−0.88272870434423567
	2	0.198	−0.70333480973846885
	2	0.185	−0.73282827159698616
	2	0.123	−0.91009488856060206
	2	0.142	−0.8477116556169435
	2	0.214	−0.66958622665080914
	2	0.077	−1.1135092748275182
	2	0.089	−1.0506099933550872
	2	0.127	−0.89619627904404309
	2	0.084	−1.0757207139381184
	2	0.731	−0.13608262304213956
	2	0.188	−0.72584215073632019
	2	0.091	−1.0409586076789064
	2	0.617	−0.20971483596675833
	2	0.893	−0.049148541111453566
	2	0.088	−1.0555173278498313
	2	0.09	−1.0457574905606752
	2	0.069	−1.1611509092627446
	2	0.191	−0.71896663275227246
	2	0.147	−0.83268266525182388
	2	0.105	−0.978810700930062
	2	0.134	−0.8728952016351923
	2	0.118	−0.92811799269387463
	2	0.1	−1

	2	0.066	−1.1804560644581312
	2	0.168	−0.77469071827413716
	2	0.218	−0.66154350639539516
	2	0.17	−0.769551078621726
	2	0.064	−1.1938200260161129
	2	1.126	0.051538390515327381
	2	0.076	−1.1191864077192086
	2	0.044	−1.3565473235138126
	2	0.069	−1.1611509092627446
	2	0.286	−0.543633966870957
	2	0.083	−1.080921907623926
	2	0.051	−1.2924298239020637
	2	0.079	−1.1023729087095586
	2	0.083	−1.080921907623926
	2	0.071	−1.1487416512809248
	2	0.081	−1.0915149811213503
	2	0.068	−1.1674910872937636
	2	0.127	−0.89619627904404309
	2	0.283	−0.54821356447570979
	2	0.097	−1.0132282657337552
	2	0.966	−0.015022873584506671
	2	0.083	−1.080921907623926
	2	0.064	−1.1938200260161129
	2	0.112	−0.9507819773298184
	2	0.053	−1.2757241303992111
	2	0.056	−1.2518119729937995
	2	0.069	−1.1611509092627446
	2	0.044	−1.3565473235138126
	2	0.081	−1.0915149811213503
	2	0.075	−1.1249387366083
	2	0.091	−1.0409586076789064
	2	0.091	−1.0409586076789064
	2	0.084	−1.0757207139381184
	2	0.044	−1.3565473235138126
	2	0.053	−1.2757241303992111
	2	0.062	−1.2076083105017461
	2	0.114	−0.94309514866352739
	2	0.09	−1.0457574905606752
	2	1.398	0.14550717140966257
	2	0.104	−0.98296666070121963
	2	0.084	−1.0757207139381184
	2	0.071	−1.1487416512809248
	2	0.094	−1.0268721464003014
	2	0.094	−1.0268721464003014
	2	0.146	−0.835647144215563
	2	0.146	−0.835647144215563
	2	0.084	−1.0757207139381184
	2	0.084	−1.0757207139381184
	2	0.134	−0.8728952016351923
	2	0.087	−1.0604807473813815
	2	0.166	−0.77989191195994489

	2	0.069	−1.1611509092627446
	2	0.063	−1.2006594505464183
	2	0.099	−1.00436480540245
	2	0.616	−0.21041928783557454
	2	0.049	−1.3098039199714864
	2	0.069	−1.1611509092627446
	2	0.064	−1.1938200260161129
	2	0.07	−1.1549019599857431
	2	0.066	−1.1804560644581312
	2	0.077	−1.1135092748275182
	2	0.099	−1.00436480540245
	2	0.099	−1.00436480540245
	2	0.059	−1.2291479883578558
	2	0.109	−0.96257350205937642
	2	0.829	−0.081445469449726471
	2	0.188	−0.72584215073632019
	2	0.104	−0.98296666070121963
	2	0.09	−1.0457574905606752
	2	0.113	−0.94692155651658028
	2	0.118	−0.92811799269387463
	2	0.071	−1.1487416512809248
	2	0.1	−1
	2	0.099	−1.00436480540245
	2	0.064	−1.1938200260161129
	2	0.076	−1.1191864077192086
	2	0.096	−1.0177287669604316
	2	0.132	−0.87942606879415008
	2	0.056	−1.2518119729937995
	2	0.091	−1.0409586076789064
	2	0.069	−1.1611509092627446
	2	0.079	−1.1023729087095586
	2	0.05	−1.3010299956639813
	2	0.602	−0.22040350874217546
	2	0.057	−1.2441251443275085
	2	0.094	−1.0268721464003014
	2	0.094	−1.0268721464003014
	2	0.115	−0.9393021596463883
	2	0.117	−0.9318141382538383
	2	0.119	−0.9244530386074693
	2	0.084	−1.0757207139381184
	2	0.066	−1.1804560644581312
	2	0.675	−0.17069622716897506
	2	0.091	−1.0409586076789064
	2	0.073	−1.1366771398795441
	2	0.143	−0.84466396253493825
	2	0.069	−1.1611509092627446
	2	0.069	−1.1611509092627446
	2	0.121	−0.91721462968354994
	2	0.066	−1.1804560644581312
	2	0.225	−0.64781748188863753
	2	0.226	−0.645891560852599

	2	0.099	−1.00436480540245
	2	0.078	−1.1079053973095197
	2	0.066	−1.1804560644581312
	2	0.068	−1.1674910872937636
	2	0.044	−1.3565473235138126
	2	0.022	−1.6575773191777938
	2	0.104	−0.98296666070121963
	2	0.078	−1.1079053973095197
	2	0.075	−1.1249387366083
	2	0.103	−0.98716277529482777
	2	0.118	−0.92811799269387463
	2	0.056	−1.2518119729937995
	2	0.081	−1.0915149811213503
	2	0.076	−1.1191864077192086
	2	0.049	−1.3098039199714864
	2	0.076	−1.1191864077192086
	2	0.101	−0.99567862621735737
	2	0.069	−1.1611509092627446
	2	0.083	−1.080921907623926
	2	0.09	−1.0457574905606752
	2	0.091	−1.0409586076789064
	2	0.104	−0.98296666070121963
	2	0.049	−1.3098039199714864
	2	0.917	−0.037630664329978893
	2	1.105	0.0433622780211295

Table S2. Individual values of 5−mC in the genomic DNA of B+ and B− females.

	Individual	Value	Log value
Female B−			
	3	0.167	−0.77728352885241669
	3	0.161	−0.79317412396815024
	3	0.087	−1.0604807473813815
	3	0.157	−0.80410034759076621
	3	0.129	−0.889410289700751
	3	0.089	−1.0506099933550872
	3	0.114	−0.94309514866352739
	3	0.084	−1.0757207139381184
	3	0.129	−0.889410289700751
	3	0.094	−1.0268721464003014
	3	0.177	−0.75202673363819339
	3	0.101	−0.99567862621735737
	3	0.176	−0.75448733218585018
	3	0.08	−1.0969100130080565
	3	0.058	−1.2365720064370627
	3	0.077	−1.1135092748275182
	3	0.088	−1.0555173278498313
	3	0.07	−1.1549019599857431
	3	0.114	−0.94309514866352739
	3	0.073	−1.1366771398795441
	3	0.059	−1.2291479883578558
	3	0.069	−1.1611509092627446
	3	0.128	−0.89279003035213167
	3	0.069	−1.1611509092627446
	3	0.075	−1.1249387366083
	3	0.063	−1.2006594505464183
	3	0.077	−1.1135092748275182
	3	0.061	−1.2146701649892331
	3	0.062	−1.2076083105017461
	3	0.133	−0.87614835903291421
	3	0.052	−1.2839966563652008
	3	0.074	−1.1307682802690238
	3	0.079	−1.1023729087095586
	3	0.832	−0.079876673709276078
	3	0.07	−1.1549019599857431
	3	0.118	−0.92811799269387463
	3	0.053	−1.2757241303992111
	3	0.083	−1.080921907623926
	3	0.083	−1.080921907623926
	3	0.062	−1.2076083105017461
	3	0.059	−1.2291479883578558
	3	0.073	−1.1366771398795441
	3	0.079	−1.1023729087095586
	3	0.135	−0.86966623150499389
	3	0.091	−1.0409586076789064
	3	0.123	−0.91009488856060206
	3	0.187	−0.72815839346350109

	3	0.106	−0.97469413473522981
	3	0.112	−0.9507819773298184
	3	0.201	−0.69680394257951106
	3	0.099	−1.00436480540245
	3	0.069	−1.1611509092627446
	3	0.066	−1.1804560644581312
	3	0.04	−1.3979400086720375
	3	0.093	−1.031517051446065
	3	0.103	−0.98716277529482777
	3	0.094	−1.0268721464003014
	3	0.91	−0.040958607678906384
	3	0.103	−0.98716277529482777
	3	0.132	−0.87942606879415008
	3	0.047	−1.3279021420642825
	3	0.063	−1.2006594505464183
	3	0.084	−1.0757207139381184
	3	0.068	−1.1674910872937636
	3	0.062	−1.2076083105017461
	3	0.079	−1.1023729087095586
	3	0.083	−1.080921907623926
	3	0.12	−0.92081875395237522
	3	0.074	−1.1307682802690238
	3	0.084	−1.0757207139381184
	3	0.066	−1.1804560644581312
	3	0.114	−0.94309514866352739
	3	0.071	−1.1487416512809248
	3	0.042	−1.3767507096020994
	3	0.084	−1.0757207139381184
	3	0.056	−1.2518119729937995
	3	0.053	−1.2757241303992111
	3	0.042	−1.3767507096020994
	3	0.053	−1.2757241303992111
	3	0.035	−1.4559319556497243
	3	0.162	−0.790484985457369
	3	0.134	−0.8728952016351923
	3	0.112	−0.9507819773298184
	3	0.13	−0.88605664769316317
	3	0.053	−1.2757241303992111
	3	0.035	−1.4559319556497243
	3	0.059	−1.2291479883578558
	3	0.107	−0.97061622231479039
	3	0.043	−1.3665315444204136
	3	1	0
	3	0.111	−0.95467702121334252
	3	0.073	−1.1366771398795441
	3	0.089	−1.0506099933550872
	3	0.066	−1.1804560644581312
	3	0.079	−1.1023729087095586
	3	0.059	−1.2291479883578558
	3	0.104	−0.98296666070121963
	3	0.099	−1.00436480540245

	3	0.04	−1.3979400086720375
	3	0.049	−1.3098039199714864
	3	0.074	−1.1307682802690238
	3	0.105	−0.978810700930062
	3	0.073	−1.1366771398795441
	3	0.064	−1.1938200260161129
	3	0.1	−1
	3	0.078	−1.1079053973095197
	3	0.081	−1.0915149811213503
	3	0.056	−1.2518119729937995
	3	0.084	−1.0757207139381184
	3	0.096	−1.0177287669604316
	3	0.078	−1.1079053973095197
	3	0.054	−1.2676062401770316
	3	0.029	−1.5376020021010439
	3	0.081	−1.0915149811213503
	3	0.084	−1.0757207139381184
	3	0.047	−1.3279021420642825
	3	0.121	−0.91721462968354994
	3	0.075	−1.1249387366083
	3	0.135	−0.86966623150499389
	3	0.785	−0.10513034325474745
	3	0.066	−1.1804560644581312
	3	0.07	−1.1549019599857431
	3	0.146	−0.835647144215563
	3	0.128	−0.89279003035213167
	3	0.042	−1.3767507096020994
	3	0.076	−1.1191864077192086
	3	0.104	−0.98296666070121963
	3	0.097	−1.0132282657337552
	3	0.098	−1.0087739243075051
	3	0.069	−1.1611509092627446
	3	0.084	−1.0757207139381184
	3	0.083	−1.080921907623926
	3	0.077	−1.1135092748275182
	3	0.07	−1.1549019599857431
	3	0.091	−1.0409586076789064
	3	0.06	−1.2218487496163564
	3	0.132	−0.87942606879415008
	3	0.06	−1.2218487496163564
	3	0.054	−1.2676062401770316
	3	0.119	−0.9244530386074693
	3	0.086	−1.0655015487564323
	3	0.097	−1.0132282657337552
	3	0.088	−1.0555173278498313
	3	0.071	−1.1487416512809248
	3	0.049	−1.3098039199714864
	3	0.118	−0.92811799269387463
	3	0.076	−1.1191864077192086
	3	0.097	−1.0132282657337552
	3	0.077	−1.1135092748275182

	3	0.086	−1.0655015487564323
	3	0.056	−1.2518119729937995
	3	0.093	−1.031517051446065
	3	0.089	−1.0506099933550872
	3	0.078	−1.1079053973095197
	3	1.226	0.088490470182396225
	3	0.079	−1.1023729087095586
	3	0.089	−1.0506099933550872
	3	0.116	−0.93554201077308152
	3	0.183	−0.73754891026957059
	3	0.069	−1.1611509092627446
	3	0.074	−1.1307682802690238
	3	0.119	−0.9244530386074693
	3	0.054	−1.2676062401770316
	3	0.104	−0.98296666070121963
	3	0.094	−1.0268721464003014
	3	0.081	−1.0915149811213503
	3	0.13	−0.88605664769316317
	3	0.098	−1.0087739243075051
	3	0.078	−1.1079053973095197
	3	0.12	−0.92081875395237522
	3	0.14	−0.85387196432176193
	3	0.075	−1.1249387366083
	3	0.135	−0.86966623150499389
	3	0.121	−0.91721462968354994
	3	0.086	−1.0655015487564323
	3	0.059	−1.2291479883578558
	3	0.125	−0.90308998699194354
	3	0.118	−0.92811799269387463
	3	0.075	−1.1249387366083
	3	0.18	−0.744727494896694
	3	0.76	−0.11918640771920865
	3	0.1	−1
	3	0.086	−1.0655015487564323
	3	0.093	−1.031517051446065
	3	0.081	−1.0915149811213503
	3	0.082	−1.0861861476162833
	3	0.089	−1.0506099933550872
	3	0.082	−1.0861861476162833
	3	0.056	−1.2518119729937995
	3	0.098	−1.0087739243075051
	3	0.116	−0.93554201077308152
	3	0.094	−1.0268721464003014
	3	0.073	−1.1366771398795441
	3	0.058	−1.2365720064370627
	3	0.075	−1.1249387366083
	3	0.055	−1.2596373105057561
	3	0.082	−1.0861861476162833
	3	0.074	−1.1307682802690238
	3	0.078	−1.1079053973095197
	3	0.084	−1.0757207139381184

	3	0.066	−1.1804560644581312
	3	0.093	−1.031517051446065
	3	0.118	−0.92811799269387463
	3	0.075	−1.1249387366083
	3	0.18	−0.744727494896694
	3	0.125	−0.90308998699194354
	3	0.069	−1.1611509092627446
	3	0.069	−1.1611509092627446
	3	0.707	−0.15058058620310061
	3	0.062	−1.2076083105017461
	3	0.061	−1.2146701649892331
	3	0.109	−0.96257350205937642
	3	0.089	−1.0506099933550872
	3	0.127	−0.89619627904404309
	3	0.104	−0.98296666070121963
	3	0.115	−0.9393021596463883
	3	0.096	−1.0177287669604316
	3	0.098	−1.0087739243075051
	3	0.119	−0.9244530386074693
	3	0.096	−1.0177287669604316
	3	0.109	−0.96257350205937642
	3	0.056	−1.2518119729937995
	3	0.111	−0.95467702121334252
	3	0.104	−0.98296666070121963
	3	0.049	−1.3098039199714864
	3	0.059	−1.2291479883578558
	3	0.123	−0.91009488856060206
	3	0.083	−1.080921907623926
	3	0.053	−1.2757241303992111
	3	0.047	−1.3279021420642825
	3	0.152	−0.81815641205522749
	3	0.178	−0.74957999769110606
	3	0.059	−1.2291479883578558
	3	0.155	−0.8096683018297085
	3	0.084	−1.0757207139381184
	3	0.081	−1.0915149811213503
	3	0.084	−1.0757207139381184
	3	0.119	−0.9244530386074693
	3	0.076	−1.1191864077192086
	3	0.125	−0.90308998699194354
	3	0.836	−0.07779372256098363
Female B+			
	4	0.085	−1.0705810742857072
	4	0.104	−0.98296666070121963
	4	0.134	−0.8728952016351923
	4	0.091	−1.0409586076789064
	4	0.182	−0.73992861201492521
	4	0.214	−0.66958622665080914
	4	0.156	−0.80687540164553839
	4	0.083	−1.080921907623926
	4	0.135	−0.86966623150499389

	4	0.079	−1.1023729087095586
	4	0.073	−1.1366771398795441
	4	0.049	−1.3098039199714864
	4	0.119	−0.9244530386074693
	4	0.115	−0.9393021596463883
	4	0.133	−0.8761483590329142
	4	0.162	−0.790484985457369
	4	0.081	−1.0915149811213503
	4	0.119	−0.9244530386074693
	4	0.74	−0.13076828026902382
	4	0.1	−1
	4	0.07	−1.1549019599857431
	4	0.176	−0.75448733218585018
	4	0.074	−1.1307682802690238
	4	0.091	−1.0409586076789064
	4	0.093	−1.031517051446065
	4	0.112	−0.9507819773298184
	4	0.063	−1.2006594505464183
	4	0.967	−0.014573525916998339
	4	0.073	−1.1366771398795441
	4	0.114	−0.94309514866352739
	4	0.148	−0.82973828460504262
	4	0.088	−1.0555173278498313
	4	0.054	−1.2676062401770316
	4	0.073	−1.1366771398795441
	4	0.069	−1.1611509092627446
	4	0.147	−0.83268266525182388
	4	0.116	−0.93554201077308152
	4	0.045	−1.3467874862246563
	4	0.131	−0.88272870434423567
	4	0.068	−1.1674910872937636
	4	0.091	−1.0409586076789064
	4	0.054	−1.2676062401770316
	4	0.164	−0.78515615195230215
	4	0.058	−1.2365720064370627
	4	0.066	−1.1804560644581312
	4	0.075	−1.1249387366083
	4	0.049	−1.3098039199714864
	4	0.057	−1.2441251443275085
	4	0.058	−1.2365720064370627
	4	0.061	−1.2146701649892331
	4	0.042	−1.3767507096020994
	4	0.667	−0.17587416608345102
	4	0.562	−0.25026368443093888
	4	0.105	−0.978810700930062
	4	0.16	−0.79588001734407521
	4	0.091	−1.0409586076789064
	4	0.057	−1.2441251443275085
	4	0.069	−1.1611509092627446
	4	0.071	−1.1487416512809248
	4	0.157	−0.80410034759076621

	4	0.058	−1.2365720064370627
	4	0.07	−1.1549019599857431
	4	0.063	−1.2006594505464183
	4	0.08	−1.0969100130080565
	4	0.14	−0.85387196432176193
	4	0.09	−1.0457574905606752
	4	0.062	−1.2076083105017461
	4	0.052	−1.2839966563652008
	4	0.059	−1.2291479883578558
	4	0.083	−1.080921907623926
	4	0.083	−1.080921907623926
	4	0.076	−1.1191864077192086
	4	0.124	−0.906578314837765
	4	0.055	−1.2596373105057561
	4	0.059	−1.2291479883578558
	4	0.085	−1.0705810742857072
	4	0.069	−1.1611509092627446
	4	0.099	−1.00436480540245
	4	0.086	−1.0655015487564323
	4	0.064	−1.1938200260161129
	4	0.669	−0.17457388223217688
	4	0.607	−0.21681130892474243
	4	0.191	−0.71896663275227246
	4	0.091	−1.0409586076789064
	4	0.097	−1.0132282657337552
	4	0.108	−0.96657624451305035
	4	0.192	−0.71669877129645043
	4	0.097	−1.0132282657337552
	4	0.121	−0.91721462968354994
	4	0.111	−0.95467702121334252
	4	0.123	−0.91009488856060206
	4	0.088	−1.0555173278498313
	4	0.131	−0.88272870434423567
	4	0.084	−1.0757207139381184
	4	0.091	−1.0409586076789064
	4	0.101	−0.99567862621735737
	4	0.984	−0.0070049015686584892
	4	0.155	−0.8096683018297085
	4	0.096	−1.0177287669604316
	4	0.061	−1.2146701649892331
	4	0.058	−1.2365720064370627
	4	0.064	−1.1938200260161129
	4	0.105	−0.978810700930062
	4	0.063	−1.2006594505464183
	4	0.105	−0.978810700930062
	4	0.096	−1.0177287669604316
	4	0.052	−1.2839966563652008
	4	0.163	−0.78781239559604221
	4	0.078	−1.1079053973095197
	4	0.074	−1.1307682802690238
	4	0.096	−1.0177287669604316

	4	0.065	−1.1870866433571443
	4	0.103	−0.98716277529482777
	4	0.085	−1.0705810742857072
	4	0.087	−1.0604807473813815
	4	0.073	−1.1366771398795441
	4	0.067	−1.1739251972991736
	4	0.049	−1.3098039199714864
	4	0.049	−1.3098039199714864
	4	0.063	−1.2006594505464183
	4	0.075	−1.1249387366083
	4	0.071	−1.1487416512809248
	4	0.081	−1.0915149811213503
	4	0.57	−0.24412514432750865
	4	0.832	−0.079876673709276078
	4	0.076	−1.1191864077192086
	4	0.09	−1.0457574905606752
	4	0.066	−1.1804560644581312
	4	0.11	−0.958607314841775
	4	0.074	−1.1307682802690238
	4	0.088	−1.0555173278498313
	4	0.089	−1.0506099933550872
	4	0.081	−1.0915149811213503
	4	0.066	−1.1804560644581312
	4	0.062	−1.2076083105017461
	4	0.084	−1.0757207139381184
	4	0.156	−0.80687540164553839
	4	0.202	−0.69464863055337622
	4	0.076	−1.1191864077192086
	4	0.068	−1.1674910872937636
	4	0.081	−1.0915149811213503
	4	0.042	−1.3767507096020994
	4	0.054	−1.2676062401770316
	4	0.069	−1.1611509092627446
	4	0.062	−1.2076083105017461
	4	0.118	−0.92811799269387463
	4	0.097	−1.0132282657337552
	4	0.081	−1.0915149811213503
	4	0.069	−1.1611509092627446
	4	0.037	−1.431798275933005
	4	0.063	−1.2006594505464183
	4	0.049	−1.3098039199714864
	4	0.068	−1.1674910872937636
	4	0.126	−0.89962945488243706
	4	0.064	−1.1938200260161129
	4	0.877	−0.057000406633959479
	4	1.232	0.090610707828406648
	4	0.076	−1.1191864077192086
	4	0.085	−1.0705810742857072
	4	0.1	−1
	4	0.09	−1.0457574905606752
	4	0.09	−1.0457574905606752

	4	0.044	−1.3565473235138126
	4	0.042	−1.3767507096020994
	4	0.139	−0.85698519974590492
	4	0.068	−1.1674910872937636
	4	0.192	−0.71669877129645043
	4	0.044	−1.3565473235138126
	4	0.084	−1.0757207139381184
	4	0.113	−0.94692155651658028
	4	0.069	−1.1611509092627446
	4	0.056	−1.2518119729937995
	4	0.094	−1.0268721464003014
	4	0.091	−1.0409586076789064
	4	0.056	−1.2518119729937995
	4	0.064	−1.1938200260161129
	4	0.064	−1.1938200260161129
	4	0.103	−0.98716277529482777
	4	0.132	−0.87942606879415008
	4	0.028	−1.5528419686577808
	4	0.106	−0.97469413473522981
	4	0.047	−1.3279021420642825
	4	0.119	−0.9244530386074693
	4	0.109	−0.96257350205937642
	4	0.109	−0.96257350205937642
	4	0.086	−1.0655015487564323
	4	0.155	−0.8096683018297085
	4	0.066	−1.1804560644581312
	4	0.814	−0.0893755951107988
	4	0.995	−0.002176919254274547
	4	0.077	−1.1135092748275182
	4	0.183	−0.73754891026957059
	4	0.069	−1.1611509092627446
	4	0.097	−1.0132282657337552
	4	0.079	−1.1023729087095586
	4	0.064	−1.1938200260161129
	4	0.056	−1.2518119729937995
	4	0.073	−1.1366771398795441
	4	0.125	−0.90308998699194354
	4	0.109	−0.96257350205937642
	4	0.153	−0.81530856918240124
	4	0.066	−1.1804560644581312
	4	0.053	−1.2757241303992111
	4	0.084	−1.0757207139381184
	4	0.161	−0.79317412396815024
	4	0.049	−1.3098039199714864
	4	0.162	−0.790484985457369
	4	0.118	−0.92811799269387463
	4	0.073	−1.1366771398795441
	4	0.066	−1.1804560644581312
	4	0.04	−1.3979400086720375
	4	0.045	−1.3467874862246563
	4	0.541	−0.26680273489343054

	4	0.684	−0.16494389827988373
